# Editorial: Microalgae for Metabolite Production Under Stress Conditions

**DOI:** 10.3389/fbioe.2021.812425

**Published:** 2022-01-06

**Authors:** Chengwei Liang

**Affiliations:** College of Marine Science and Biological Engineering, Qingdao University of Science and Technology, Qingdao, China

**Keywords:** microalga, microalga biomass, high-value added products, stress condition, molecular mechanism

Microalgae are found in marine and freshwater environments and convert carbon dioxide into potential biofuels (carbohydrates, lipids), foods and high-value products such as lutein, chlorophyll, and zeaxanthin driven by solar energy, normally under certain stressful cultivation conditions. The past 20 years have seen a flood of patents related to microalgal biofuels as shown in the article by Li et al. Briefly, the majority of patents are related to strains and their cultivation under different conditions, especially some stress conditions, the introduction of regulating reagents, the combination of different downstream treatments and bio-refinery methods or processes. In general, there are no natural microalgae suitable for high value-added production directly. Therefore, in the last 2 decades, the artificial modification of natural strains is concerning, especially with the development of gene engineering.

In most instances, the accumulation of high value-added products occurs under stress conditions, which adversely affects the growth of microalgae. Thus, the industrial production of microalgae may have been limited by biomass supply. Therefore, studying the mechanism of accumulation of high additive products under stress conditions may solve this problem. In this field of research, several articles reported the molecular mechanism of microalgae from different aspects. Xi et al. reported that reactive oxygen species may serve as mediators or second messengers to trigger the fine-tuning of key genes that are involved in photosynthesis and β-carotene biosynthesis in *Dunaliella salina* under stress conditions. The changes in photosynthesis efficiency and energy metabolism will be useful for β-carotene accumulation in *D. salina* under stress conditions. Wang et al. examined six transcription factors (TFs) belonging to the MYB, MYB_related, NF-YC, Nin-like, and C3H families involved in the transcription regulation of 27 astaxanthin synthesis-related genes according to the regulatory network which will help to understand the transcription regulatory mechanism of astaxanthin synthesis in *Haematococcus pluvialis* under stress conditions. These TFs could affect astaxanthin synthesis by directly regulating the β-carotene ketolase gene (CrtO) which is the key gene involved in astaxanthin biosynthesis. Polyunsaturated fatty acids (PUFAs) compositions in serine/threonine kinases (STKs) gene spkD and spkG knockout mutant *Synechocystis* sp. PCC6803 are lower than those in the wild-type, which showed that STKs play important roles in regulating PUFA biosynthesis in *Synechocystis* sp. PCC6803 (Chen et al.). These results may also have implications for other algae.

In the past decades, various cultivation strategies including heterotrophic cultivation, photoautotrophy-to-heterotrophy cultivation and heterotrophy-to-photoautotrophy cultivation modes in addition to photoautotrophic cultivation have emerged to enhance microalgal lipid production in order to balance the costs. Heterotrophy-to-photoautotrophy cultivation takes advantages of both cultivation modes, which produce high-quality biomass via heterotrophy and induce biosynthesis of particular end-products under photoautotrophic conditions. Hu Zhang et al. studied the mechanisms underlying the enhanced biomass and lipid production of *Scenedesmus acuminatus* during trophic transition from heterotrophy to photoautotrophy under stress conditions (high light and N limited conditions). Carbon metabolism was deduced to provide sufficient energy to sustain vigorous growth of *S. acuminatus* cells under stress. Enhanced lipid production may be attributable to the upregulation of acetyl-CoA carboxylase (ACCase) and diacylglycerol acyltransferase 2(DGAT2)at the gene expression level.

Microalgae-bacteria co-cultivation systems are another new avenue that can be investigated to improve the mass production of microalgae. Ouyang et al. employed metabolomics to gain a better understanding of the underlying causes of improvements in growth and paramylon production in *Euglena gracilis* when co-cultivated with *Vibrio natriegens*. Some differential metabolites including economically important metabolites such as choline, ectoine, 4R-aminopentanoic acid, methyl N-methylanthranilate and methyl carbamate were found in the co-cultivation group which may provide clues for the industrial application of *E. gracilis*.

More knowledge on the molecular mechanisms of high-value products in microalgae can guide utilization of key molecules to solve the problems that limit the commercial application of microalgae. In this area of research, the chloroplast glyceraldehyde-3-phosphate dehydrogenase (cGAPDH) overexpressed strain *Chlamydomonas reinhardtii* P3-GAPDH reported in the article (Zhu et al.) demonstrated higher carbon fixation and PUFA synthesis efficiency. The overexpression of chloroplast GAPDH gene enabled the P3-GAPDH to maintain high photosynthetic activity and promote biomass production, i.e., the carbohydrate and lipid content increased by 96.6 and 93.4%, respectively, under normal cultivation conditions. Thus, it is hoped that this will lead to simultaneous high production of biomass and energy storage compounds.

Microalgae are also good organisms for bioremediation applications due to their strong acclimation to changing metal ion concentrations in the environment via metal homeostasis. Metabolomics and proteomic analysis were used in two studies (He et al. and Zhen et al.), to examine the mechanism of acclimatization of microalgae to metal ions. The results showed that in order to meet the intracellular requirements of ions, metals can be transferred into the cell via the uptake system and macromolecular metabolites such as proteins, pigments, and lipids were regulated to adapt to stress conditions.

Microalgae have great application potential as they can accumulate high-value products under stress conditions. However, stress conditions usually suppress the growth of microalgae which limit their commercial application. The information on this topic will provide clues to achieve two-win between the production of high microalgal biomass and high value compounds.

